# Imprinting disorder in donor cells is detrimental to the development of cloned embryos in pigs

**DOI:** 10.18632/oncotarget.20390

**Published:** 2017-08-22

**Authors:** Xuexiong Song, Fangzheng Li, Zhongling Jiang, Yueping Sun, Huatao Li, Shansong Gao, Liping Zhang, Binghua Xue, Guimin Zhao, Jingyu Li, Zhonghua Liu, Hongbin He, Yanjun Huan

**Affiliations:** ^1^ College of Veterinary Medicine, Qingdao Agricultural University, Qingdao, Shandong Province, China; ^2^ College of Life Science, Northeast Agricultural University, Harbin, Heilongjiang Province, China; ^3^ College of Life Science, Shandong Normal University, Jinan, Shandong Province, China

**Keywords:** imprinting, donor cell, somatic cell nuclear transfer, cloned embryo, pig

## Abstract

Imprinting disorder during somatic cell nuclear transfer usually leads to the abnormality of cloned animals and low cloning efficiency. However, little is known about the role of donor cell imprinting in the development of cloned embryos. Here, we demonstrated that the imprinting (H19/Igf2) in porcine fetus fibroblasts derived from the morphologically abnormal cloned fetuses (the abnormal imprinting group) was more hypomethylated, and accordingly, significantly higher H19 transcription and lower Igf2 expression occurred in comparison with those in fibroblasts derived from morphologically normal cloned fetuses (the normal imprinting group) or donor fetus fibroblasts (the control group). When these fibroblasts were used as donor cells, the abnormal imprinting group displayed an even lower imprinting methylation level, in correspondence to the significantly downregulated expression of Dnmt1, Dnmt3a and Zfp57, and a markedly reduced blastocyst rate, while the normal imprinting group took on the similar patterns of imprinting, gene expression and embryo development to the control group. When 5-aza-dC was applied to reduce the fibroblasts imprinting methylation level in the normal imprinting group, cloned embryos displayed the more severely impaired imprinting and significantly lower blastocyst rate. While the upregulated H19 transcription in the abnormal imprinting group was knocked down, the imprinting statuses were partly rescued, and the cleavage and blastocyst rates significantly increased in cloned embryos. In all, donor cell imprinting disorder reduced the developmental efficiency of cloned embryos. This work provides a new insight into understanding the molecular mechanism of donor cells regulating the cloned embryo development.

## INTRODUCTION

Somatic cell nuclear transfer (SCNT) has achieved in many species, owning a broad application prospect in the basic research, agriculture, biomedicine, etc [1]. However, the overall cloning efficiency remains low, and the developmental abnormalities frequently occur, limiting the wide application of cloning technology [2, 3].

It is generally believed that the developmental abnormalities of cloned animals and low cloning efficiency are largely due to the imprinting disorder [4]. Imprinting is an epigenetic regulatory mechanism to ensure a monoallelic parental-specific expression pattern and the normal growth and development of embryos [5]. Then, the imprinting disorder would alter the expression patterns of imprinted genes, resulting in the poor embryo development. Thus, imprinting has been considered as an excellent model to evaluate the developmental efficiency of cloned embryos.

Naturally, genomic imprinting is erased and established during gametogenesis and faithfully maintained throughout the subsequent embryo development in the normal reproduction [5]. Indeed, during early embryogenesis, genomic imprinting is recognized and protected by the specific DNA binding complexes including Dnmt1, Zfp57 and Trim28, et al., to resist the global DNA demethylation and remethylation [6]. As for animal cloning, SCNT bypasses the progress of imprinting erasure and establishment, seeming that just the imprinting maintenance could support the cloned embryo development, however, cloned embryos suffers imprinting defects [7], indicating that the imprinting maintenance mechanism is destroyed in cloned embryos, and SCNT may establish or maintain the wrong imprinting, resulting in the poor cloning efficiency.

At present, H19/Igf2, representing genomic imprinting, is widely studied and critical for the normal embryo development [4]. Igf2 paternally expresses and acts as a growth factor, while the transcription of H19, a long noncoding RNA, is maternal. The parent-specific expression of H19/Igf2 is controlled by the differentially methylated region 3 (DMR3, widely accepted) of H19 imprinting control region (ICR). The DMR3 is methylated on the paternal allele, then the enhancer element prefers Igf2 paternal expression. On the maternal allele, H19 transcription has a cis silencing effect on the adjacent Igf2 expression. This mechanism allows for the precise control of H19 and Igf2 expression [8]. During the assisted reproduction, H19/Igf2 imprinting hypomethylation usually occurs, and the upregulated H19 transcription leads to the developmental defects [9]. As for the semi-cloning, H19 DMR deletion in androgenetic haploid embryonic stem cells can efficiently support the full-term development of semi-cloned embryos [10]. And, the developmental failure of uniparental embryos also reveals the indispensable role of gametic H19/Igf2 imprinting in the normal embryo development [11]. Then, it is wondered whether the H19/Igf2 imprinting in donor cells, just like in gametes, regulates the cloned embryo development. As the hypomethylated H19/Igf2 imprinting usually occurs during SCNT, and the methylation status of donor cell lines can also affect the cloned embryo development [12–14], then, it is speculated that a close relationship between H19/Igf2 imprinting status in donor cells and the cloned embryo development must exist, needing to be clarified.

Numerous studies have demonstrated that H19/Igf2 imprinting problems constrain the cloning efficiency [15–17], and our previous studies also revealed that the retarded development of cloned embryos and fetuses was associated with the H19/Igf2 imprinting disorder, and alteration of donor cell DNA methylation impairs the cloned embryo development could be due to the disrupted H19/Igf2 imprinting in donor cells [4, 18]. Thus, in this study, the role of donor cell imprinting in the development of cloned embryos was investigated. Our results demonstrated that when fibroblasts derived from the morphologically abnormal cloned fetus with the hypomethylated H19/Igf2 imprinting were used as donor cells, cloned embryos displayed a markedly reduced development and an even more severely impaired H19/Igf2 imprinting, and downregulation of H19/Igf2 methylation level in normal imprinting PFFs by 5-aza-dC also resulted in the significantly lower blastocyst rate. While H19 transcription was knocked down in abnormal imprinting group, H19/Igf2 imprinting status and the cloned embryo development were obviously improved. This work would have important implications in improving the cloning efficiency.

## RESULTS

### Disrupted imprinting in fibroblasts derived from the morphologically abnormal cloned fetuses

**Figure 1:**
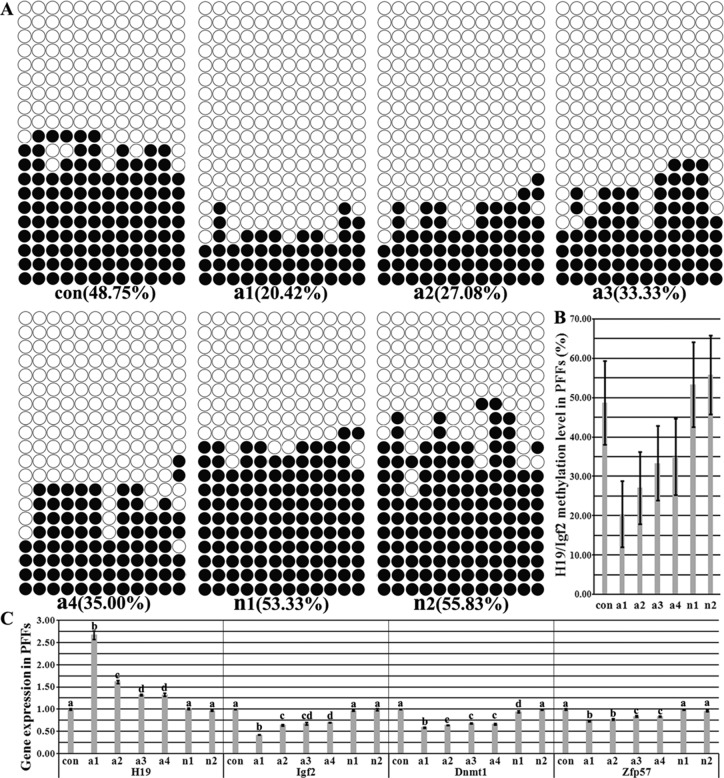
The methylation statuses and transcription of H19/Igf2 imprinting and the expression of genes related to the imprinting methylation maintenance in PFFs (**A**) the methylation statuses of H19/Igf2 imprinting in PFFs derived from *in vivo* fertilized and cloned fetuses, (**B**) H19/Igf2 methylation levels in PFFs derived from *in vivo* fertilized and cloned fetuses, (**C**) the transcription of H19/Igf2 and genes related to the imprinting methylation maintenance in PFFs derived from *in vivo* fertilized and cloned fetuses. PFFs derived from the morphologically abnormal cloned fetuses displayed the hypomethylated H19/Igf2 imprinting, upregulated H19 transcription and downregulated expression of Igf2 and imprinting methylation maintenance related genes. con represented PFFs derived from the *in vivo* fertilized fetuses, a1, a2, a3 and a4 represented PFFs derived from the morphologically abnormal cloned fetuses, and n1 and n2 represented PFFs derived from the morphologically normal cloned fetuses, respectively. Black or white circles indicate methylated or unmethylated CpG sites, respectively. ^a–d^Values for a given gene with different superscripts differ significantly (*P* < 0.05).

Our previous study has shown that 4 of 6 porcine cloned fetuses were morphologically abnormal and their imprinting levels were hypomethylated [4]. Here, the methylation statuses and transcription of H19/Igf2 and the expression of genes related to the imprinting methylation maintenance were further detected in PFFs (porcine fetus fibroblasts) derived from the morphologically abnormal and normal cloned fetuses (Figure 1 and Supplementary Figures A1, A2, A3, A4). The results demonstrated that the imprinting of PFFs (Supplementary Figures A1, A2, A3, A4) derived from the morphologically abnormal cloned fetuses (A1, A2, A3 and A4) was hypomethylated (20.42%, 27.08%, 33.33% and 35.00%, respectively), and accordingly, the significantly upregulated H19 transcription and downregulated Igf2 expression occurred in comparison with those of PFFs (Supplementary Figures N1 and N2) derived from the morphologically normal cloned fetuses (Supplementary Figures N1 and N2) and donor PFFs. And more, the expression levels of Dnmt1 and Zfp57 also significantly decreased in PFFs (Supplementary Figures A1, A2, A3, A4). While no significant differences of H19/Igf2 imprinting and the expression of Dnmt1 and Zfp57 were observed between PFFs (Supplementary Figures N1 and N2) and donor PFFs. Thus, imprinting was disrupted in fibroblasts derived from the morphologically abnormal cloned fetuses.

### Imprinting disorder in donor cells reduced the developmental efficiency of cloned embryos

**Figure 2:**
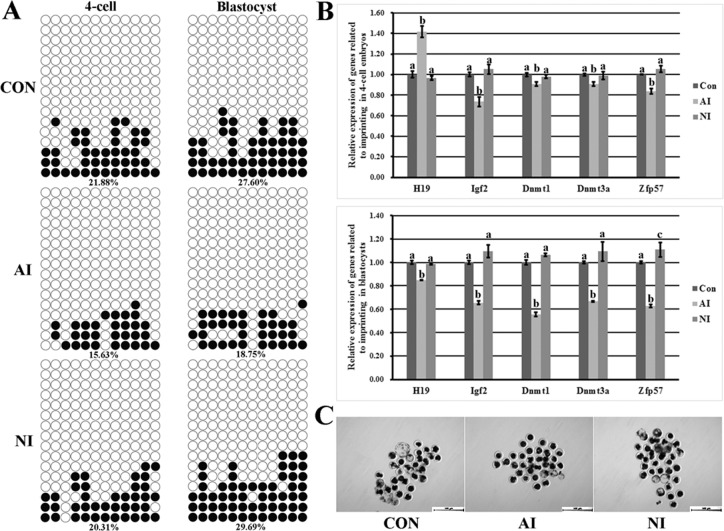
The methylation statuses and transcription of H19/Igf2 imprinting and the expression of genes regulating imprinting methylation in cloned embryos and the cloned blastocysts derived from the control, abnormal imprinting and normal imprinting PFFs (**A**) the methylation statuses of H19/Igf2 imprinting at the 4-cell and blastocyst stages of cloned embryos derived from the control, abnormal imprinting and normal imprinting PFFs, (**B**) the transcription of H19/Igf2 and genes regulating imprinting methylation at the 4-cell and blastocyst stages of cloned embryos derived from the control, abnormal imprinting and normal imprinting PFFs, (**C**) the cloned blastocysts derived from the control, abnormal imprinting and normal imprinting PFFs (Scale *bar* = 500 μm). The cloned embryos derived from the abnormal imprinting PFFs displayed the hypomethylated H19/Igf2 imprinting, disrupted H19 transcription, reduced expression of Igf2 and genes regulating imprinting methylation and downregulated blastocyst rate. CON, AI and NI represented cloned embryos derived from the control, abnormal imprinting and normal imprinting PFFs, respectively. Black or white circles indicate methylated or unmethylated CpG sites, respectively. ^a–c^Values for a given gene with different superscripts differ significantly (*P* < 0.05).

To investigate whether donor cell imprinting plays a critical role in the development of cloned embryos, PFFs a2 (not a1, due to the poor cell proliferation, Supplementary Figure 2A and Supplementary Figure 3), representing the abnormal imprinting group, PFFs n2, as the normal imprinting group, and donor PFFs, namely the control group, were employed. Here, compared with the control group, the abnormal imprinting group displayed an even lower imprinting methylation level (15.63% vs 21.88% in 4-cell embryos and 18.75% vs 27.60% in blastocysts, respectively, Figure 2A), the significantly higher H19 transcription at the 4-cell stage but interestingly and obviously lower H19 expression in blastocysts and the significantly reduced expression of Igf2, Dnmt1, Dnmt3a and Zfp57 in cloned embryos (Figure 2B), and a markedly reduced blastocyst rate was also observed (12.24% vs 20.08%, *P* < 0.05, Figure 2C and Table 1). For the normal imprinting group, the imprinting status, gene expression and embryo development were similar to those in the control group (Figure 2). Taken together, these results suggested that serial nuclear transfer did not improve the cloning efficiency and imprinting disorder in donor cells was detrimental to the cloned embryo development.

**Table 1:** Development of cloned embryos derived from donor cells with the various imprinting statuses

Group	No. embryos (Rep.)	No. embryos fused (% ± SEM)	No. embryos cleaved (% ± SEM)^#^	No. blastocysts (% ± SEM)^#^	Blastocyst cell numbers (mean ± SEM)^&^
Control	221 (5)	174 (78.79 ± 2.35)	151 (86.94 ± 1.34)	35 (20.08 ± 0.95)^a^	36 ± 2 (*n* = 34)
Abnormal imprinting	225 (5)	163 (72.55 ± 1.61)	133 (81.62 ± 2.07)	20 (12.24 ± 0.85)^b^	34 ± 2 (*n* = 17)
Normal imprinting	227 (5)	175 (77.04 ± 1.93)	149 (85.36 ± 2.02)	40 (22.75 ± 1.14)^a^	37 ± 3 (*n* = 39)

^#^Cleavage and blastocyst rates were adjusted for fusion rates.

^&^Blastocyst cell numbers, less than 16, were not included.

^a–b^Values in the same column with different superscripts differ significantly (*P* < 0.05).

### Donor cell imprinting hypomethylation induced by 5-aza-dC led to the poor developmental efficiency of cloned embryos

**Figure 3:**
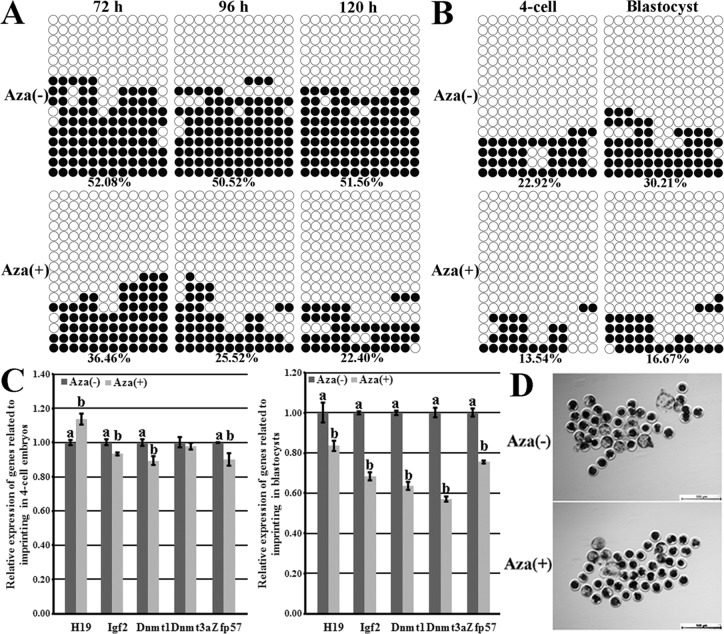
H19/Igf2 imprinting methylation statuses and transcription and the expression of genes regulating imprinting methylation in PFFs and cloned embryos and the cloned blastocysts derived from PFFs treated with 5-aza-dC (**A**) the methylation statuses of H19/Igf2 imprinting in PFFs treated with 5-aza-dC, (**B**) the methylation statuses of H19/Igf2 imprinting at the 4-cell and blastocyst stages of cloned embryos derived from PFFs treated with 5-aza-dC, (**C**) the transcription of H19/Igf2 and genes regulating imprinting methylation at the 4-cell and blastocyst stages of cloned embryos derived from PFFs treated with 5-aza-dC, (**D**) the cloned blastocysts derived from PFFs treated with 5-aza-dC (Scale *bar* = 500 μm). Treating donor cells with 5-aza-dC resulted in the hypomethylated H19/Igf2 imprinting in PFFs and cloned embryos, disrupted H19 transcription, reduced expression of Igf2 and genes regulating imprinting methylation and downregulated blastocyst rate. Aza (+) and Aza (−) represented PFFs treated with 5-aza-dC or not. Black or white circles indicate methylated or unmethylated CpG sites, respectively. ^a–b^Values for a given gene with different superscripts differ significantly (*P* < 0.05).

To investigate whether imprinting hypomethylation in donor cells was the cause of the poor cloned embryo development in the abnormal imprinting group, 5-aza-dC was employed to reduce the donor cell imprinting methylation level in the normal imprinting group, then, the imprinting statuses, the expression patterns of genes related to the imprinting methylation maintenance and the cloned embryo development were examined. After PFFs were treated with 5-aza-dC, the obviously downregulated imprinting methylation levels were observed (36.46% vs 52.08% at 72 h, 25.52% vs 50.52% at 96 h, and 22.40% vs 51.56% at 120 h, respectively, Figure 3A) in comparison with those in PFFs untreated, and treating PFFs for 96 h did not markedly affect the cell proliferation (Supplementary Figure 2B) and was further applied in the Aza (+) group. When these treated PFFs were used as donor cells, compared with those in the Aza (−) group, the greatly lower imprinting methylation levels (13.54% vs 22.92% in 4-cell embryos and 16.67% vs 30.21% in blastocysts, respectively, Figure 3B), the significantly upregulated H19 transcription in 4-cell embryos but downregulated H19 expression in blastocysts (Figure 3C), the markedly reduced expression of Igf2, Dnmt1, Dnmt3a and Zfp57 in cloned embryos (Figure 3C) and the significantly lower blastocyst rate (15.40% vs 22.95%, *P* < 0.05, Figure 3D and Table 2) occurred in the Aza (+) group. Thus, imprinting hypomethylation in donor cells could be detrimental to the cloned embryo development.

**Table 2:** Development of cloned embryos derived from the normal imprinting donor cells treated with 5-aza-dC

Group	No. embryos (Rep.)	No. embryos cleaved (% ± SEM)	No. blastocysts (% ± SEM)
Aza (−)	135 (5)	118 (87.76 ± 1.39)	28 (22.95 ± 1.96)^a^
Aza (+)	157 (5)	127 (82.19 ± 1.65)	20 (15.40 ± 0.61)^b^

^a–b^Values in the same column with different superscripts differ significantly (*P* < 0.05).

### H19 knockdown in the abnormal imprinting donor cells was beneficial for the cloned embryo development

**Figure 4:**
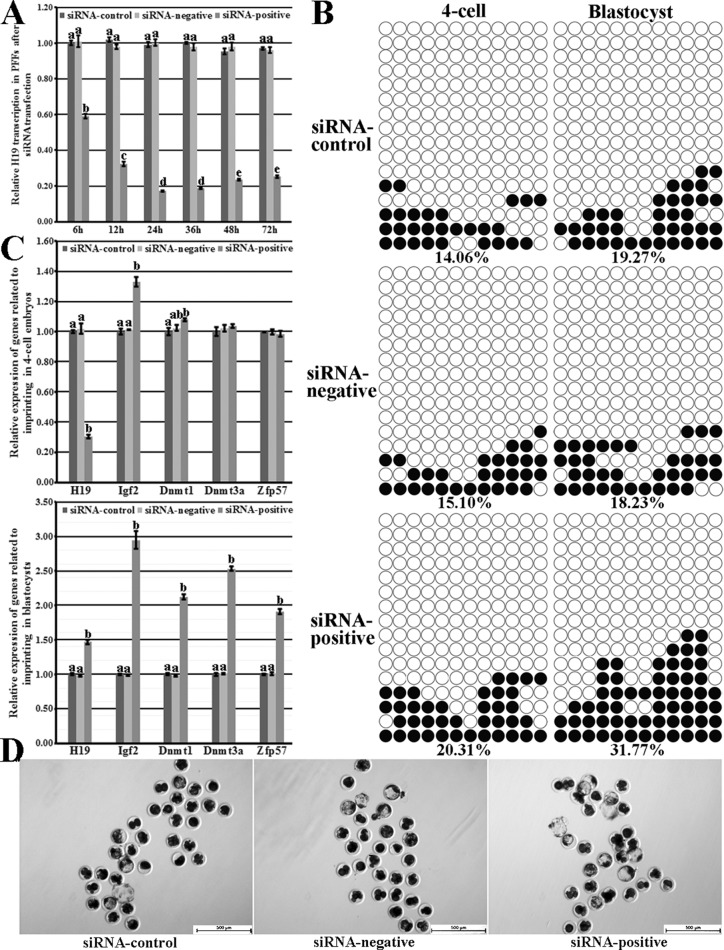
H19/Igf2 imprinting transcription and methylation statuses and the expression of genes regulating imprinting methylation in PFFs and cloned embryos, and the cloned blastocysts derived from the abnormal imprinting PFFs with H19 knockdown (**A**) H19 transcription in the abnormal imprinting PFFs after siRNA transfection, (**B**) the methylation statuses of H19/Igf2 imprinting at the 4-cell and blastocyst stages of cloned embryos derived from the abnormal imprinting PFFs in the siRNA-control, siRNA-negative and siRNA-positive groups, (**C**) the transcription of H19/Igf2 and genes regulating imprinting methylation at the 4-cell and blastocyst stages of cloned embryos derived from the abnormal imprinting PFFs in the siRNA-control, siRNA-negative and siRNA-positive groups, (**D**) the cloned blastocysts derived from the abnormal imprinting PFFs in the siRNA-control, siRNA-negative and siRNA-positive groups (Scale *bar* = 500 μm). H19 knockdown in the abnormal imprinting PFFs improved H19/Igf2 imprinting methylation and transcription, the expression of genes regulating imprinting methylation and blastocyst rate. siRNA-control, siRNA-negative and siRNA-positive represented the abnormal imprinting PFFs transfected with none siRNA, negative siRNA and positive siRNA, respectively. Black or white circles indicate methylated or unmethylated CpG sites, respectively. ^a^–cValues for a given gene with different superscripts differ significantly (*P* < 0.05).

To further investigate whether the upregulated H19 transcription in donor cells resulted in the poor cloned embryo development, siRNA was employed to reduce H19 expression in the abnormal imprinting group. When siRNA was transfected into PFFs a2, no significant decrease of cell number during PFFs culture occurred (Supplementary Figure 2C), and H19 transcription was significantly knocked down (59.00%, 32.41%, 17.19%, 18.88%, 23.54% or 25.19% at 6 h, 12 h, 24 h, 36 h, 48 h or 72 h in the siRNA-positive group vs 100.02% at 6 h in the siRNA-control group, respectively, *P* < 0.05, Figure 4A). Then, donor cells with H19 knockdown for 24 h were used for SCNT, and the siRNA-positive group took on the upregulated imprinting methylation levels (20.31% vs 14.06% or 15.10% in 4-cell embryos and 31.77% vs 19.27% or 18.23% in blastocysts, respectively, Figure 4B) in comparison with the siRNA-control or siRNA-negative group, suggesting that H19 knockdown in donor cells could rescue the impaired imprinting in cloned embryos. Responding to the ameliorated imprinting, the significantly downregulated H19 transcription in 4-cell embryos and the upregulated Igf2 expression in cloned embryos were observed, and the expression levels of Dnmt1, Dnmt3a and Zfp57 in blastocysts were significantly higher in the siRNA-positive group compared with the siRNA-control or siRNA-negative group (*P* < 0.05, Figure 4C). Interesting, the significantly higher H19 transcription in blastocysts was also observed in the siRNA-positive group, seemingly inconsistent with its imprinting methylation status. Notably, compared with the siRNA-control or siRNA-negative group, the siRNA-positive group displayed the significantly upregulated cleavage and blastocyst rates (87.28% vs 78.82% or 79.32% for the cleavage rate, and 27.90% vs 12.85% or 11.34% for the blastocyst rate, respectively, *P* < 0.05, Figure 4D and Table 3). Thus, H19 knockdown in abnormal imprinting donor cells was beneficial for the cloned embryo development.

**Table 3:** Development of cloned embryos derived from the abnormal imprinting donor cells with H19 knockdown

Group	No. embryos (Rep.)	No. embryos cleaved (% ± SEM)	No. blastocysts (% ± SEM)
siRNA-control	171 (5)	135 (78.82 ± 1.91)^a^	22 (12.85 ± 1.37)^a^
siRNA-negative	184 (5)	146 (79.32 ± 1.73)^a^	21 (11.34 ± 1.28)^a^
siRNA-positive	173 (5)	151 (87.28 ± 1.85)^b^	48 (27.90 ± 1.84)^b^

^a–b^Values in the same column with different superscripts differ significantly (*P* < 0.05).

## DISCUSSION

It is known that abnormal imprinting results in the low cloning efficiency [19]. In this study, our results displayed that the imprinting status in PFFs derived from the morphologically abnormal cloned fetuses was disordered, and these PFFs led to the severely impaired imprinting in cloned embryos and the markedly downregulated blastocyst rate. The poor development of cloned embryos derived from PFFs with the hypomethylated imprinting induced by 5-aza-dC also demonstrated that the imprinting disruption in donor cells reduced the development of cloned embryos. Additionally, the result that H19 knockdown in donor cells with the hypomethylated imprinting improved the cloned embryo development further supported that donor cell imprinting status could determine the cloning efficiency. Thus, the developmental efficiency of cloned embryos was closely associated with the donor cell imprinting status.

Generally, imprinting disorder results in the developmental abnormalities of cloned animals and low cloning efficiency [4]. Here, we demonstrated that the imprinting status in PFFs derived from the morphologically abnormal cloned fetuses was disrupted, coinciding with the previous report [20]. Notably, along with the smaller sizes of the abnormal cloned fetuses, the even lower methylation levels and more disrupted expression patterns of imprinting genes were observed. Whereas, no obvious differences were observed between the morphologically normal cloned group and the *in vivo* fertilized group. Collectively, these results further support the view that imprinting defect leads to the poor cloned embryo development [19]. It is known that imprinting can be faithfully retained by the maintenance enzymes including Dnmt1, Zfp57 and Trim28, etc [6]. Here, our study displayed that the significantly reduced expression of Dnmt1 and Zfp57 occurred in PFFs derived from the morphologically abnormal cloned fetuses, suggesting that the key molecules for genomic imprinting methylation maintenance was lost during the cloned embryo development, thereby resulting in the imprinting hypomethylation and the upregulated H19 transcription, further leading to the retarded cloned fetuses. Moreover, increasing studies also display that the retarded development of cloned fetuses is due to the aberrant imprinting [4, 14]. Accordingly, the perturbed imprinting could be the cause of the morphological abnormality of cloned fetuses.

Previous studies have demonstrated that imprinting disruption is the cause of parthenogenetic or androgenetic embryo developmental failure, revealing that gametic imprinting is indispensable for the normal embryo development [11, 21]. Here, when PFFs with the abnormal imprinting were used as donor cells, along with the decreased expression levels of the imprinting methylation maintenance genes, the imprinting status was severely disrupted in cloned embryos, probably leading to the reduced development of cloned embryos. And, no significant differences of the imprinting status and embryo development were observed between the normal imprinting group and the control group, further suggesting that the error information in donor cells could be inherited to the cloned embryos and impair the embryo development.

Encouragingly, the regulation of genomic imprinting can rescue the failed development of parthenogenetic and semi-cloned embryos [22, 23], then, alteration of donor cell imprinting can further identity the concrete relationship between the donor cell imprinting status and the cloned embryo development. When donor cell imprinting methylation level was reduced by 5-aza-dC, the hypomethylated imprinting occurred in cloned embryos and the blastocyst rate was significantly downregulated, further supporting the previous reports that treating donor cell with 5-aza-dC cannot enhance the cloned embryo development [24, 25]. H19 is a key regulator of the imprinted gene network [26]. The hypomethylated imprinting of H19/Igf2 leads to the high H19 transcription, and H19 biallelic expression has been reported in cloned embryos [14], then, it is speculated that the upregulated H19 expression in donor cells can be the cause of the low cloning efficiency, as the upregulated H19 transcription occurred in the abnormal imprinting group and the 5-aza-dC treatment group. Expectedly, our results demonstrated that H19 knockdown in the abnormal imprinting group partly rescued the disrupted imprinting and enhanced the cloned embryo development. These improvements may be due to that H19 could interact with genes responsible for the imprinting methylation establishment and maintenance, and the relatively normal expression of Dnmt1, Zfp57 and Dnmt3a in cloned embryos can help explain this view. Certainly, numerous molecules can regulate genomic imprinting, and, more information is needed to clarify the imprinting regulatory mechanism during the cloned embryo development [8]. Taken together, donor cell imprinting status is critical for the cloned embryo development.

Indeed, the low development of cloned embryos is associated with the imprinting disruption, and the imprinting status in the individual embryo at the same stage, even if it can develop to the blastocyst or fetus, could be not all the same, suggesting that the imprinting status could also be different among the donor cells. In this study, only the imprinting status of the grouped fibroblasts not the real donor cells was examined to reveal the role of donor cell imprinting in the cloned embryo development, as it is impossible to detect the imprinting status in the individual donor cell that gives rise to its corresponding cloned embryo, then, the culture of cell colony derived from one single somatic cell and single cell bisulfite sequencing will be adopted to examine the precise role of donor cell imprinting regulating the cloned embryo development in the further study [27]. The imprinting data of cloned embryos was also based on dozens of pooled embryos, and whether imprinting is maintained or erased then reestablished in the normal cloned embryo development is still unclear, thus, embryo biopsy and single cell methylation and RNA sequencing will be further applied to reveal the real imprinting methylation status dynamics and the clear molecule regulatory mechanism of donor cell imprinting regulating SCNT mediated reprogramming [28, 29].

In conclusion, our results demonstrated that the imprinting status in PFFs derived from the morphologically abnormal cloned fetuses was aberrant, and these PFFs led to the even severely disrupted imprinting and reduced cloned embryo development. And, donor cell imprinting hypomethylation induced by 5-aza-dC was detrimental to the cloned embryo development, while H19 knockdown in the hypomethylated imprinting donor cells enhanced the cloned embryo development. These investigations suggest that donor cell imprinting can regulate the cloned embryo development and provide a new insight into improving the cloning efficiency and the health of cloned animals.

## MATERIALS AND METHODS

Chemicals were purchased from Sigma-Aldrich Corporation (St. Louis, MO, USA), and disposable and sterile plasticware was obtained from Nunclon (Roskilde, Denmark), unless otherwise stated. All experiments were approved by the Animal Care Commission of Qingdao Agricultural University according to animal welfare laws, guidelines and policies. All surgery was performed under sodium pentobarbital anaesthesia, and all efforts were made to minimize suffering.

### Donor cell culture

Donor cell culture has been described previously [25]. Briefly, porcine *in vivo* fertilized or cloned fetuses were obtained from sows after anaesthetized and sacrificed at day 35 of pregnancy, then PFFs were isolated from the fetuses under sodium pentobarbital anaesthesia. After the removal of fetal head, internal organs and limbs, the remaining tissues were finely minced into pieces, digested with 0.25% trypsin-0.04% ethylenediaminetetraacetic acid solution (GIBCO), and dispersed in high glucose enriched Dulbecco's modified Eagle's medium (DMEM, GIBCO) containing 10% fetal bovine serum (FBS, GIBCO) and 1% penicillin-streptomycin (GIBCO). The dispersed cells were centrifuged, resuspended and cultured in DMEM. Until confluence, PFFs were digested, centrifuged, resuspended in FBS containing 10% dimethyl sulfoxide and stored in liquid nitrogen until use. Prior to SCNT, PFFs were thawed, cultured and used in 2–3 passages.

### Donor cell treatment

For 5-aza-dC treatment [25], PFFs were cultured in DMEM supplemented with 10 nM (the optimal concentration) 5-aza-dC without any antibiotics for 72 h, 96 h or 120 h, respectively.

For H19 knockdown, methods of siRNA design, synthesis and transfection have been reported in our study [3]. According to the requirement of Invitrogen Block-iT RNAi Designer and H19 mRNA information, the stealth siRNA was designed and synthesized, and the sequence was CCTCCTAGCTCTGACTCAAGAATAT. The negative sequence was CCTTAGCTCTGACTCAAGAACCTAT. Then, siRNAs were dissolved with Rnase free H_2_O to the concentration of 20 μM. Before transfection, PFFs were cultured in 400 μl Opti-MEM (GIBCO), 1.5 μl Lipofectamine 2000 (Lipo 2000, Invitrogen) was added into 50 μl Opti-MEM and incubated at room temperature for 5 min, 20 μM H19 siRNA was diluted into 500 nM with Opti-MEM, and, 100 μl siRNA-Lipo 2000 complexes was obtained through a mixture of 50 μl Opti-MEM with 500 nM siRNA and 50 μl Opti-MEM with 1.5 μl Lipo 2000, incubated at room temperature for 30 min and added into each 24-well culture plate with PFFs and 400 μl Opti-MEM. After 6 h, the medium including siRNA-Lipo 2000 complexes was replaced by DMEM containing 10% FBS and 1% penicillin-streptomycin. The interference efficiency was examined at 6 h, 12 h, 24 h, 36 h, 48 h or 72 h posttransfection, respectively. The negative siRNA with the same amount was transfected as a control.

After 5-aza-dC treatment or H19 siRNA transfection, PFFs were harvested at 6 h, 12 h, 24 h, 36 h, 48 h, 72 h, 96 h or 120 h, respectively, and the cell number at every time point was determined with a hemacytometer. Then, the manner of 5-aza-dC treatment with the obvious imprinting hypomethylation or siRNA transfection with H19 significant knockdown but no notable effect on cell proliferation was applied in the subsequent experiment.

### Oocyte *in vitro* maturation

Oocyte maturation has been reported [30]. Briefly, porcine ovaries were collected from a local slaughterhouse. Just after exposure, ovaries were placed into physiological saline with antibiotics at 37°C and transported to the laboratory. Follicles were aspirated, and follicular contents were washed with HEPES-buffered Tyrode's lactate. Cumulus-oocyte complexes (COCs) were recovered and cultured in maturation medium. After 42 h, COCs were vortexed in hyaluronidase for 30 sec to remove cumulus cells. Only oocytes with the visible polar body, regular morphology and homogenous cytoplasm were used.

### SCNT, embryo development and collection

The procedure for SCNT has been described in our reports [25, 31]. Briefly, matured oocytes and donor cells were placed into manipulation medium. After oocyte enucleation, donor cells were placed into the perivitelline space. Fusion and activation of the cell-cytoplast complexes were induced by electroporation. Then, the reconstructed embryos were cultured in porcine zygote medium-3 (PZM-3) for the subsequent development, and the cleavage and blastocyst rates were evaluated at 48 h and 156 h postactivation, respectively. For the collection of cloned embryos, 4-cell and blastocyst embryos in each group were collected at 48 h and 156 h, respectively.

### Nuclear staining

For blastocyst cell number, cloned embryos at 156 h postactivation were treated with acidic Tyrode's solution to remove zona pellucida, fixed in 4% paraformaldehyde for 30 min, and stained with 10 μg/ml Hoechst 33342 for 5 min in the dark. After staining, cloned blastocysts were washed and mounted on slides. Then, blastocyst cell number was examined under ultraviolet light from a fluorescence microscope.

### Quantitative real-time PCR

Measurement of gene expression with quantitative real-time PCR has been applied in our studies [30, 32]. Briefly, total RNA was extracted from 10^4^ PFFs or 50 pooled embryos at each stage using an RNeasy Micro Kit (Qiagen) according to the manufacturer's instructions, and the elution volume was 50 μl. Reverse transcription was performed using a PrimeScript RT Reagent Kit (TaKaRa). The 100 μl reaction volume contained 20 μl 5 × PrimeScript Buffer, 5 μl PrimeScript RT Enzyme Mix I, 5 μl Oligo dT Primer (50 μM), 5 μl Random 6 mers (100 μM), 50 μl Total RNA and 15 μl RNase Free dH_2_O. The reaction condition was 37°C for 15 min and 85°C for 5 sec, and the cDNA was stored at −20°C until use. For quantitative real-time PCR, reactions were performed in 96-well optical reaction plates (Applied Biosystems) using SYBR Premix ExTaq II (TaKaRa) and a 7500 Real-Time PCR System (Applied Biosystems). Each reaction mixture (20 μl) contained 2 μl cDNA solution, 10 μl 2 × SYBR Premix Ex Taq II, 1.6 μl PCR primers (10 μM), 0.4 μl ROX Reference Dye II (50×) and 6 μl dH_2_O. Thermal cycling conditions were 95°C for 30 sec, 40 two-step cycles of 95°C for 5 sec and 60°C for 34 sec, and finally a dissociation stage consisting of 95°C for 15 sec, 60°C for 1 min and 95°C for 15 sec. For each sample, the cycle threshold (CT) values were obtained from three replicates. The primers used for the amplification of target and internal reference genes were presented in Supplementary Table 1. The relative expression levels of target genes were analyzed using the 2^−ΔΔCT^ method.

### Bisulfite sequencing

Bisulfite sequencing has been reported [33]. Briefly, pooled samples were treated with sodium bisulfite to convert all unmethylated cytosine to uracil using an EZ DNA Methylation-Direct^TM^ Kit (Zymo Research) according to the manufacturer's protocol. For PFFs, a Universal Genomic DNA Extraction Kit (TaKaRa) was used to extract genomic DNA. For samples of 50 4-cell or 10 blastocyst stage pooled zona pellucida-removed cloned embryos in each group, digestion was performed in M-Digestion Buffer plus with Proteinase K at 50°C for 20 min. After treatment, a cytosine to thymine conversion was carried out at 98°C for 10 min and 64°C for 2.5 h. Then, the samples were desalted, purified and diluted with M-Elution Buffer. Subsequently, nested PCR was carried out to amplify DMR3 of H19/Igf2 using the previously reported primers as described in Supplementary Table 2 and Hot Start Taq^TM^ Polymerase (TaKaRa) with a profile of 94°C for 5 min, 40 cycles of 94°C for 30 sec, 55°C for 30 sec and 72°C for 1 min, followed by 72°C for 10 min. Products from the first amplification reaction were used in the second PCR reaction, and the optimal annealing temperature of inner primers was 50°C. Then, the amplified products were verified by electrophoresis and purified using an Agarose Gel DNA Purification Kit (TaKaRa), and the purified fragments were cloned into a pMD18-T Vector (TaKaRa) and subjected to sequence analysis.

### Statistical analysis

Differences in data (mean ± SEM) were analyzed with the SPSS statistical software. Statistical analyses of data concerning embryo development, gene expression and cell proliferation were performed with one-way ANOVA or *t*-test when two groups were compared. For all analyses, differences were considered to be statistically significant when *P* < 0.05.

## SUPPLEMENTARY MATERIALS FIGURES AND TABLES



## Abbreviations

SCNT: somatic cell nuclear transfer
DMR: differentially methylated region
ICR: imprinting control region
PFFs: porcine fetal fibroblasts
FBS: fetal bovine serum
DMEM: Dulbecco's modified Eagle's medium
COCs: cumulus-oocyte complexes
CT: cycle threshold
PZM-3: porcine zygote medium-3.
