# Supplementary material for: Imprinting disorder in donor cells is detrimental to the development of cloned embryos in pigs
Source: Oncotarget. 2017 Aug 22;8(42):72363–74. doi: 10.18632/oncotarget.20390 (PMC5641136; doi:10.18632/oncotarget.20390)
Supplement: Supplementary file 1 [file oncotarget-08-72363-s001.pdf]

Imprinting disorder in donor cells is detrimental to the development of cloned embryos in pigs

SUPPLEMENTARY MATERIALS

Supplementary Table 1: Detail of primers for quantitative real-time PCR

Gene	Primer sequence (5′-3′)	Length (bp)	Accession number
H19	F: TCAAACGACAAGAGATGGTGCTA<br>R: CAAGTAGTGTAGTGGCTCCAGAATG	118	AY_044827
Igf2	F: CCGTGGCATCGTGGAAGAGTG<br>R: TCCAGGTGTCATAGCGGAAGAAC	170	NM_213883
Dnmt1	F: GCGTCTTGCAGGCTGGTCAGTA<br>R: CTTCTTATCATCGACCACGACGCT	152	NM_001032355
Dnmt3a	F: ATGTGGTTCGGAGACGGCAAGT<br>R: GCTCTCGTCGTTGTCATGGCA	195	NM_001097437
Zfp57	F: GGAGACCAAGTCCGACTCACAT<br>R: CAGCGGTTGGACTGCTCTATG	141	NM_001123132
β2m	F: CTTTCTACCTTCTGGTCCACACTG<br>R: GTGGTCTCGATCCCACTTAACTATC	116	NM_213978
18s	F: AATCTCGGGTGGCTGAACGC<br>R: CCGTTCTTAGTTGGTGGAGCGAT	143	NR_002170

Supplementary Table 2: Detail of primers for H19/Igf2 imprinting bisulfite sequencing

Gene	Primer sequence (5′-3′)	Primer annealing temperature (°C)	Length (bp)	Accession number
H19/Igf2	Outer<br>F: GGTTTTAGGGGGATATTTTTT<br>R: TTAAAAAAACATTACTTCCATATAC	55	384	AY_044827
	Inner<br>F: GATTTTTAGGTTTGTTATTATTT<br>R: CAAATATTCAATAAAAAAACCC	50	208	

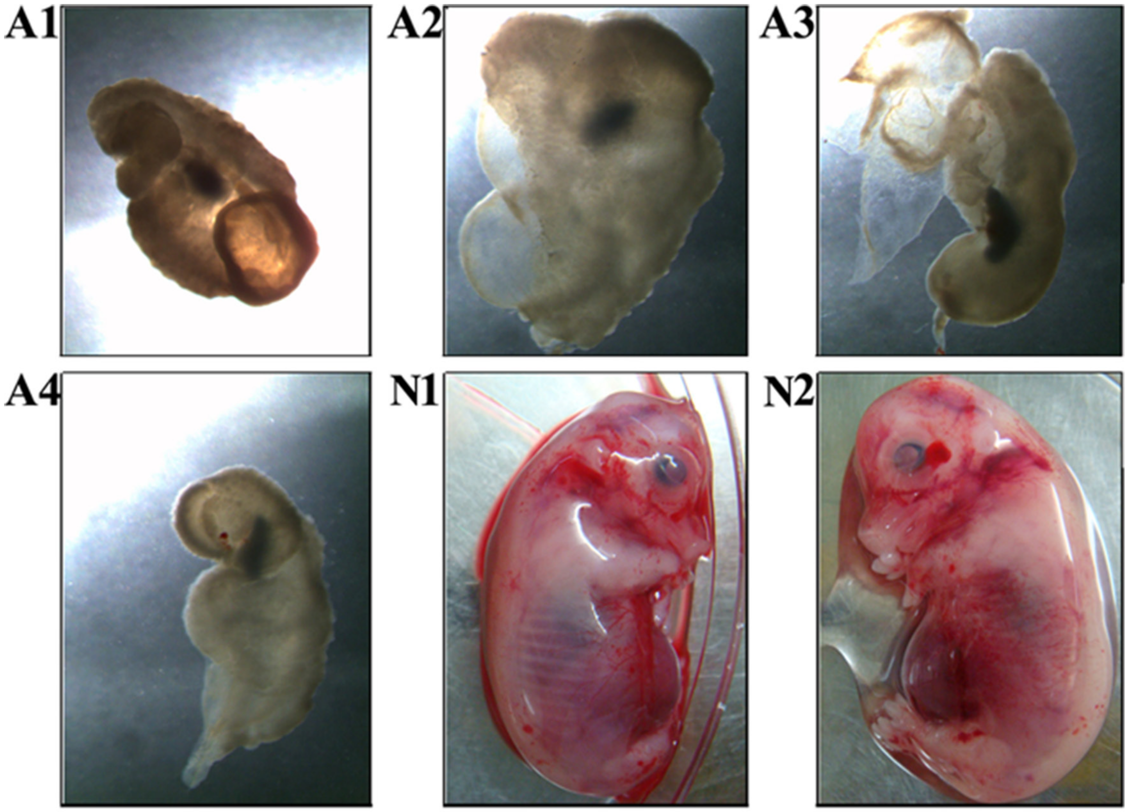

Supplementary Figure 1: The morphologies of 35-day cloned fetuses. **A1**, **A2**, **A3** and **A4** represent the morphologically abnormal cloned fetuses, and **N1** and **N2** represent the morphologically normal cloned fetuses. In comparison with the morphologically normal cloned fetuses, the morphologically abnormal cloned fetuses displayed the obviously smaller sizes.

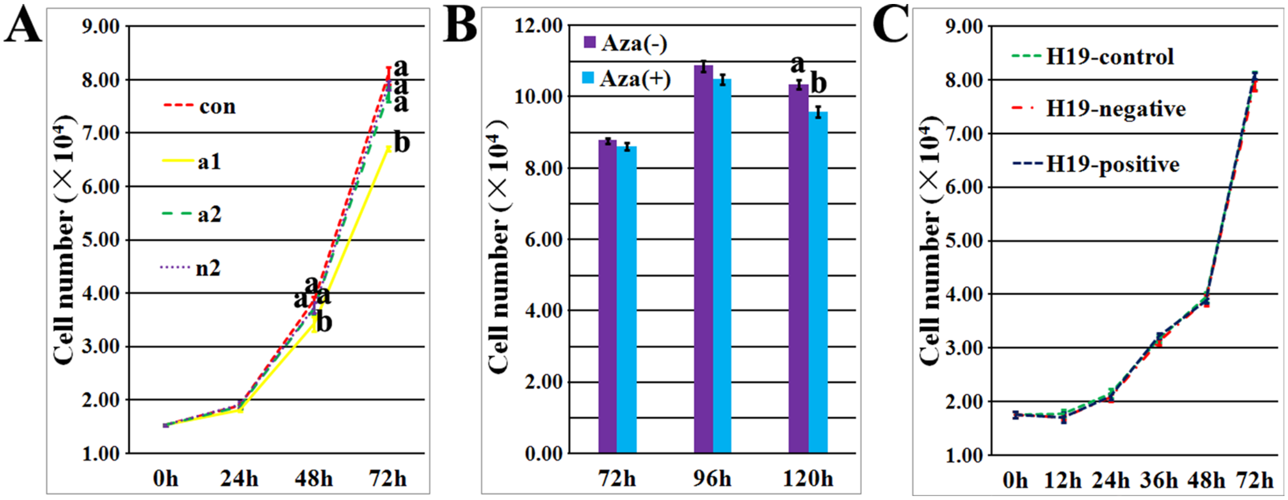

Supplementary Figure 2: Cell proliferation of PFFs with different treatments. (**A**) cell proliferation of PFFs derived from the in vivo fertilized, morphologically abnormal cloned and morphologically normal cloned fetuses. (**B**) cell proliferation of the normal imprinting PFFs treated with 5-aza-dC. (**C**) cell proliferation of the abnormal imprinting PFFs after H19 knockdown. Only PFFs with no significant change of cell proliferation after different treatments were used for the subsequent somatic cell nuclear transfer. con, a1 (2) and n2 represent PFFs derived from the in vivo fertilized, morphologically abnormal cloned and morphologically normal cloned fetuses, respectively. Aza (+) represents PFFs treated with 5-aza-dC. H19-control, H19-negative and H19-positive represent PFFs transfected with none siRNA, negative siRNA and positive siRNA, respectively. The data were expressed as mean ± SEM. $^{a\text{–}b}$Values at a certain time point with different superscripts differ significantly ($P < 0.05$).

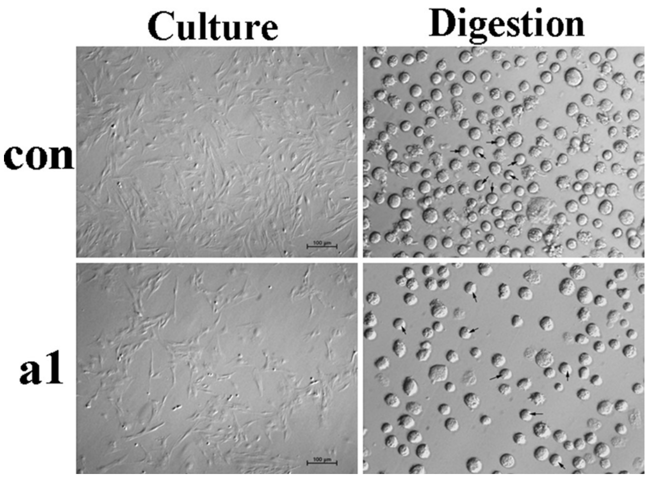

Supplementary Figure 3: Cell morphologies of PFFs under culture and digestion. con and a1 represent PFFs derived from the *in vivo* fertilized and morphologically abnormal cloned fetuses. a1 displayed the poor cell proliferation.
